# Sarcopenia Is Associated with Cognitive Impairment and Depression in Elderly Korean Women

**Published:** 2018-03

**Authors:** Inhwan LEE, Jinkyung CHO, Haeryun HONG, Youngyun JIN, Donghyun KIM, Hyunsik KANG

**Affiliations:** College of Sport Sciences, Sungkyunkwan University, Suwon, Republic of Korea

**Keywords:** Women, Sarcopenia, Mental health, Risk factors, Aging

## Abstract

**Background:**

Sarcopenia has been implicated in the increased risk for cognitive impairment and depression associated with aging. In this cross-sectional study, we investigated the relationship of sarcopenia with cognitive impairment (MCI) and depression in a sample of 201 community-dwelling Korean women (mean age of 74.0±6.8 yr) between 2014 and 2015.

**Methods:**

The Korean version of mini-mental state examination and the center for epidemiologic studies depression scale was used to assess cognitive performance and depression, respectively. Grp strength was measured with a dynamometer. Demographics, body composition, education, alcohol consumption, and history of cardiovascular diseases were assessed as covariates.

**Results:**

Odds ratio (OR) and 95% confidence interval (95% CI) of MCI and depression were calculated according to sarcopenia status. Compared to non-sarcopenic, pre-sarcopenic and sarcopenic women had the ORs of 2.160 (95% CI of 0.840 – 5.554, *P*=0.030) and of 5.493 (95% CI of 1.854 – 16.270, *P*=0.002) for MCI. The OR of pre-sarcopenia for MCI remained significant (*P*=0.030) even after adjustments for age, body mass index (BMI), lean body mass, and education, while the OR of sarcopenia for MCI was no longer significant (*P*=0.084) when adjusted for the covariates. Compared to non-sarcopenic, pre-sarcopenic and sarcopenic women had the ORs of 3.750 (95% CI of 1.137 – 12.370, *P*=0.030) and of 4.687 (95% CI of 1.127–19.505, *P*=0.034) for depression. The ORs of pre-sarcopenia and sarcopenia for depression remained statistically significant (*P*=0.020 and *P*=0.042, respectively) even after adjusted for the covariates

**Conclusion:**

Sarcopenia was significantly associated with MCI and depression in otherwise healthy community dwelling elderly Korean women.

## Introduction

The population aged 60 yr and older has been increased in nearly all countries as a consequent of decreased mortality and declining fertility (1). While the majority of older adults remain mentally healthy, many are at risk of developing neuropsychiatric disorders such as dementia and depression (2). Older adults with depressive symptoms are at increased risk for developing cognitive impairments (3). In fact, cognitive impairment often accompanies depression or vice versa, but their relationship is bi-directional and complex such that it remains an important research topic in the elderly (4).

Prevalence of sarcopenia, defined as a progressive decline in muscle mass, strength, and function (5), increases with aging, in conjunction with physical inactivity (6), malnutrition (7) and higher body fat (8). Since those modifiable risk factors are associated with cognitive impairment and depression, sarcopenia may be linked to the pathological processes that lead to the mental disorders in older adults. This hypothesis has been supported by a recent cross-sectional study showing that sarcopenia may increase the risk of cognitive decline and depression in Taiwanese older adults (9).

South Korea is moving toward an aged society at the fastest pace in the world. In 2010, the Korean population aged 65 yr and older reached 11%, and the number is projected to increase to 24.3% in 2030 and reach 37.4% in 2050 (10). In a nationwide survey of South Koreans aged 65 yr and older, prevalence of sarcopenia was 9.7% for men and 11.8% for women (11). Another nationwide survey reported that prevalence of dementia was 8.1% (95% CI=6.9–9.2) for overall dementia and 24.1% (95% CI = 21.0–27.2) for MCI (12). In addition, prevalence of major depressive disorders was estimated to range from 3.7% to 11.0% (13). The majority of sufferers (14.9%∼37.0%) were over 60 yr with higher prevalence in women than in men (13).

Low body mass index (BMI) in the elderly was associated with the pathological severity of Alzheimer’s disease upon postmortem examination (14) as well as more rapid onset of cognitive impairment (15), suggesting that weight loss may precede the onset of cognitive impairment leading to mild cognitive impairment and/or dementia. Aging features lean body mass loss in conjunction with sarcopenia, contributing to cognitive impairment and brain atrophy (16, 17). However, there is contradictory evidence for the relationship of sarcopenia to cognitive decline and depression in the Korean population. In a longitudinal cohort from the Ansan Geriatric (AGE) study, depression was significantly associated with low body mass and sarcopenia in elderly Korean men (18). In the 2010–2011 Korean National Health and Nutrition Examination Survey, however, no association was found between sarcopenia and depression in Korean adults aged 20 yr and older (19). Cognitive decline was significantly associated with frailty components such as grip strength and walking speed in elderly Korean women (20).

Further studies are necessary to establish whether sarcopenia is a modifiable risk factor for both cognitive decline and depression in elderly Korean adults. In a cross-sectional design, this study examined the relationship between sarcopenia status, mild cognitive impairment and depression symptoms in community-dwelling South Korean women aged 65 yr and older.

## Methods

### Participants

Overall, 223 participants were initially recruited via advertisements (i.e., local newspapers and flyers) in local community centers in the Northwestern Gyeonggi Province, South Korea between Dec 2014 and Dec 2015. Eligibility criteria included: women aged 65 yr or older; no self-reported difficulty walking; no difficulty performing basic activities of daily living (ADL); no reported cane use, walker, crutches or other special equipment to get around; no active treatment for cancer in the prior three years; and no enrollment in a lifestyle intervention trial.

After receiving information describing the study, potential participants were screened for the eligibility criteria described above. Of the 223 initial participants, 17 were excluded due to mobility limitations and/or use of cane, walker, and crutches. Additional 5 participants refused to participate in body composition and/or physical activity assessments due to personal reason(s). Consequently, 201 participants were available for analyses.

### Procedures

Data were collected during two separate visits. During the first visit, demographic characteristics and general health status were assessed with a standardized self-administered questionnaire modified from ACSM’s Guidelines for Exercise Testing and Prescription (21) and geriatric nurses conducted interviews.

During the second visit, body weight and height were measured with a stadiometer attached to a scale while subjects wore a hospital gown and foam slippers (Jenix, Seoul, Korea). BMI was calculated (kg/m^2^) from height and weight. The mini-mental state examination (MMSE) and the center for epidemiologic studies depression scale (CES-D) were administered. Gait speed, hand-grip strength, and muscle mass were measured to assess sarcopenia status as described below. Informed consent was obtained from all subjects prior to study participation. The Sungkyunkwan University Institutional Review Board, in accordance with the World Medical Association Declaration of Helsinki, approved the study protocol.

### Determination of sarcopenia

Sarcopenia was evaluated based on the diagnostic criteria of the Asia Working Group for Sarcopenia (AWGS) recommendations (22). Low muscle mass (LMM) was defined as height-adjusted skeletal muscle mass of <5.4 kg/m^2^, measured with a whole-body DEXA scanner using the Lunar DPX Pro total body scan mode (GE Lunar, Madison, WI, USA). Low muscle strength (LMS) was defined as handgrip strength of <18 kg, assessed with a digital dynamometer (TTM-YD, Tokyo, Japan). Low physical performance (LPP) was defined as gait speed of <0.8 m/s, assessed with a 6-meter walk test (OptoGait System, Bolzano, Italy). Individuals with only LMM were categorized as pre-sarcopenic; those with LMM plus LMS or LPP as sarcopenic; those with higher muscle mass regardless of LMS or LPP were categorized as non-sarcopenic (23).

#### Determination of mild cognitive impairment

Global cognitive function was assessed with the MMSE. This MMSE version was validated for Korean elderly (24). The MMSE measures elder cognitive status by quantifying orientation (10 points), short-term memory and recall (6 points), attention (5 points), phonic fluency (2 points), ability to follow verbal commands (4 points), judgment (2 points) and ability to copy a double pentagon (1 point) (25). MMSE scores range between 0 and 30. Higher scores indicate better cognitive status. The most widely accepted and frequently used cutoff score for the MMSE is 23, with scores of 23 or lower indicating the presence of mild cognitive impairment (MCI).

#### Determination of depression

The CES-D 20-item version was used (26, 27). This test was designed to identify depression symptoms and estimate their severity. Higher scores indicate greater depression. The cut-off score for probable depression was 16. The cutoff score for definite depression was 25.

### Demographic and potential covariates

Age, educational background, alcohol consumption, and history of cardiovascular or cerebrovascular disease, were assessed and included as covariates. A detailed medical history, including these variables, was obtained from the participants or their informants. Cardiovascular disease was defined as present if the participant had a history of coronary artery disease, myocardial infarction, atrial fibrillation or other arrhythmias, heart valve disease, or congestive heart failure. Cerebrovascular disease was defined as present if the participant had a history of hemorrhagic or ischemic stroke or transient ischemic attacks.

### Statistical analyses

All variables were checked for normality and subjected to log10 transformation, if necessary, before statistical analyses. Tests for linear trends in frequency and means of sample characteristics according to incremental severity of sarcopenia status were performed with the chi-square test and linear contrast one-way of ANOVA, respectively. Odds ratio (OR) and 95% confidence interval (95% CI) for MCI and depression status according to incremental severity of sarcopenia status were estimated using logistic regression before and after adjustment for the covariates measured in this study. Alpha was set at *P*=0.05. SPSS statistical software, ver. 21.0 (Chicago, IL, USA) was used to perform all statistical analyses.

## Results

There was a significant positive, linear trend in mean age (*P*<0.001), and negative linear trends in education (*P*=0.002), BMI (*P*=0.008), and lean body mass (*P*<0.001) across incremental sarcopenia severity from non-sarcopenia to sarcopenia. Significant negative and linear trends were found in ASM index (*P*<0.001), gait speed (*P*<0.001), and handgrip strength (*P*<0.001) across incremental sarcopenia severity. In addition, significant negative and linear trends were found in MMSE (*P*<0.001) and CES-D (*P*=0.050) scores across incremental sarcopenia severity (Table 1). Compared to non-sarcopenic (OR=1), pre-sarcopenic and sarcopenic women had ORs of 2.632 (*P*=0.035) and 5.493 (*P*=0.002), respectively, for MCI. When adjusted for age, BMI, lean body mass, and educational background, the OR of pre-sarcopenia for MCI was substantially attenuated but remained significant (*P*=0.030), while the OR of sarcopenia for MCI was no longer significant (*P*=0.084).

**Table 1:** Description of measured parameters according to the incremental severity of sarcopenia status

***Variables***	***Total (N=201)***	***Non-sarcopenia (n=91/45%)***	***Pre-sarcopenia (n=84/42%)***	***Sarcopenia (n=26/13%)***	***P for linear trends***
**Demographic Measures**					
Age (yr)	74.3±6.6	72.2±6.7	75.9±5.8	76.6±6.5	< .001
Post menopause (yr)	49.4±5.3	50.0±5.5	49.1±4.5	48.8±6.4	.537
Educational background (yr)	6.1±4.1	7.2±3.9	5.1±4.1	5.7±3.9	.002
**Body Composition**					
BMI (kg/m^2^)	24.9±3.4	25.2±3.3	25.0±3.1	22.9±3.6	.008
Body fat (%)	35.3±6.2	34.8±6.1	36.1±5.8	34.9±7.5	.336
Lean body mass (kg)	34.7±3.4	35.8±2.8	34.4±3.4	31.6±3.2	< .001
Waist circumference (cm)	86.7±10.3	85.8±9.1	87.6±8.0	84.1±10.4	.153
**AWGS definition of Sarcopenia**					
ASM index (kg/m^2^)	5.8±0.6	6.1±0.5	5.8±0.5	5.0±0.3	< .001
Gait speed (m/s)	1.1±0.3	1.2±0.2	1.1±0.3	1.1±0.2	< .001
Hand grip strength (kg)	18.7±4.6	21.8±2.5	16.4±4.8	15.0±2.4	< .001
**MMSE score**	24.6±4.2	25.9±3.3	23.5±4.6	23.6±4.2	< .001
^a^**CES-D score**	8.8±9.9	6.4±7.4	10.3±10.7	12.2±12.7	.050
**CVD risk factors, n (%)**					.647
1	15 (7.5%)	6 (6.6)	7 (8.3)	2 (7.7)	
2	39 (19.4%)	21 (23.1)	12 (14.3)	6 (23.1)	
≥3	147 (73.1%)	64 (70.3)	65 (77.4)	18 (69.2)	
**Medication, n (%)**					.412
0	32 (16.0%)	13 (14.3)	15 (17.9)	4 (15.4)	
1	68 (33.8%)	30 (33.0)	26 (31.0)	12 (46.1)	
2	63 (31.3%)	34 (37.3)	25 (29.8)	4 (15.4)	
≥3	38 (18.9%)	14 (15.4)	18 (21.3)	6 (23.1)	
**Alcohol consumption, n (%)**					.692
0	91 (45.3%)	39 (42.9)	42 (50.0)	10 (38.5)	
1–2	97 (48.3%)	44 (48.3)	38 (45.2)	15 (57.7)	
3–5	6 (3.0%)	3 (3.3)	2 (2.4)	1 (3.8)	
≥5	7 (3.4%)	5 (5.5)	2 (2.4)	0 (0)	

ASM: appendicular skeletal muscle mass; MMSE-DS: mini mental state examination of dementia screening; CES-D: the center for epidemiologic studies depression scale; CVD: cardiovascular disease; AWGS: the Asia Working Group for Sarcopenia.

a Log_10_ transformed CES-D scores were used for statistical analyses, and its raw scores were presented in the table.

Compared to non-sarcopenic (OR=1), pre-sarcopenic and sarcopenic women had ORs of 3.750 (*P*=0.030) and 4.687 (*P*=0.034), respectively, for depression. When adjusted all the covariates, the ORs of pre-sarcopenia and sarcopenia for depression remained statistically significant (*P*=0.022 and *P*=0.042, respectively) (Table 2).

**Table 2:** Odds ratios of mild cognitive impairment and depression according to the incremental severity of sarcopenia status

***Levels of sarcopenia***	***OR of MCI (95% C.I.)***		***OR of depression (95% C.I.)***	
	Unadjusted	Adjusted^*^	Unadjusted	Adjusted^*^
Non-sarcopenia	1	1	1	1
Pre-sarcopenia	2.632 (1.070–6.474)	2.361 (0.914–6.009)	3.750 (1.137–12.370)	4.519 (1.239–16.496)
Sarcopenia	5.493 (1.854–16.270)	4.502 (1.317–15.319)	4.687 (1.127–19.505)	5.448 (1.063–27.920)

For the adjusted ORs, age, BMI, lean body mass, and education were entered as covariates in the logistic regression analyses // OR: odds ratio; CI: confidence interval; MCI: mild cognitive impairment

## Discussion

In this cross-sectional design, we investigated the relationship of sarcopenia status with MCI and depression in community-dwelling Korean elderly women. Pre-sarcopenia and sarcopenia showed prevalence of 42% and 13%, respectively.

Consistent with previous findings (28), sarcopenia severity increased linearly with age and decreased linearly with BMI, lean body mass, and education. Higher BMI and lean body mass may reflect better nutrition as well as regular physical activity including exercise, which contributes to prevent/delay the onset of sarcopenia with aging. In addition, educational background was found to be another factor in determining sarcopenia associated with aging, but a definite explanation cannot be given due to the cross-sectional nature of this study.

In particular, we found that AWGA-based sarcopenia severity was inversely and significantly associated with MMSE and CES-D scores, suggesting its potential role as a determinant of cognitive impairment as well as depression in Korean older women. The current finding is consistent with previous studies of Asian populations such as Korean, Japanese, and Taiwanese older adults. In a prospective study (29), sarcopenia was an independent predictor of cognitive decline in community-dwelling Japanese older adults. Similarly, sarcopenia was significantly associated with cognitive impairment in community-dwelling older Taiwanese (30). Compared to controls, adults aged 40 yr or older with sarcopenia were more likely to have both cognitive and physical impairments (31). Further, changes in body composition were closely related to cognitive impairment (32). In the Epidemiology de osteoporosis (EPIDOS) study, however, sarcopenia was not shown to be associated with cognitive impairment in elderly women (33). Similarly, neither changes in body composition nor gait speed was not associated with cognitive dysfunction in a 7-yr follow-up study of 181 women aged 75 yr or older (34).

In addition to cognitive function, we also found that sarcopenia was significantly associated with depression. Consistent with this finding, depression was negatively correlated with BMI in women and with low body mass and sarcopenia in Korean men (35). Similarly, obese elderly Korean women were less likely to suffer from depression, as compared to those with apparently normal weight (18). On the other hand, no associations were found between sarcopenia and depression prevalence or depression symptoms in Korean adults (19). As an explanation for these conflicting reports, the CES-D used in this study aimed to measure the depression symptomatology spectrum rather than collecting only severity level information relevant to diagnosis or clinical assessment, implying that the association between sarcopenia and depression may not be completely established in this study.

Overall, we found that sarcopenia was significantly associated with both cognitive impairment and depression symptoms in Korean elderly women. Although further investigation is required to explore the interrelationship of sarcopenia, cognitive impairment and depressive symptoms in older adults, several possible explanations can be given to the relationship of sarcopenia with cognitive impairment and depression. First, sarcopenia associated with aging often leads to physical inactivity secondary to low muscle mass, strength, and function in conjunction with poor diet (36). Second, a common mechanism shared by sarcopenia, cognitive impairment, and depression was inflammation. Age-related chronic low-grade inflammation is associated with elevations in circulating interleukin-6 and tumor necrosis factor-α, and they have been reported as a common factor for the occurrence of sarcopenia as well as and the development of cognitive impairment and depression (37). Finally, oxidative stress related to chronic diseases may cause wasting of skeletal muscle in conjunction with neuronal degeneration and cognitive impairment (38). Collectively, these conditions might result in depressive symptoms and/or depression.

This study had several limitations. First, only ambulatory volunteers were surveyed and measured. Despite the 75% response rate and locally designated examination centers, the prevalence of sarcopenia, MCI, and depression might be underestimated, partially due to the relatively small sample size of the current study. Second, there are still some associated factors not adequately evaluated in this study. For example, nutrition and physical activity, which are two major modulators associated with sarcopenia and normal aging, should be accounted for in a future study using a large sample size. Third, the cross-sectional nature of this study did not allow us to pinpoint a causal relationship between sarcopenia, cognitive function, and depression symptoms. A prospective and/or randomized controlled study will be required to explore the causal relationship between sarcopenia, cognitive impairment and depression symptoms in older Korean adults.

## Conclusion

Sarcopenia was significantly and positively associated with cognitive impairment and depression in otherwise healthy community-dwelling elderly Korean women, suggesting that a healthy lifestyle consisting of good nutrition and physical activity/fitness should be promoted as a means to prevent cognitive impairment and/or depression symptoms as well as sarcopenia with normal aging.

## Ethical considerations

Ethical issues (Including plagiarism, informed consent, misconduct, data fabrication and/or falsification, double publication and/or submission, redundancy, etc.) have been completely observed by the authors.
